# Evaluating the clinical significance of tumor-expressed C-reactive protein in chromophobe renal cell carcinoma

**DOI:** 10.1038/s41598-026-60322-2

**Published:** 2026-07-14

**Authors:** Marie Mikuteit, Stefanie Zschäbitz, Michael Autenrieth, Wilko Weichert, Arndt Hartmann, Sandra Steffens, Franziska Erlmeier

**Affiliations:** 1 Department for Dermatology and Allergy, Hannover Medical School, Carl-Neuberg-Str. 1, 30625 Hannover, Germany; 2 Department of Medical Education, Hannover Medical School, Hannover, Germany; 3 Department of Medical Oncology, National Center of Tumor Diseases, University Hospital Heidelberg, 69120 Heidelberg, Germany; 4 Department of Urology, Technical University of Munich, Klinikum Rechts Der Isar, München, Germany; 5 Institute for Pathology and Pathological Anatomy, Technical University Munich, Munich, Germany; 6 Institute of Pathology, University Hospital of Erlangen, Erlangen, Germany; 7 Department for Rheumatology and Immunology, Hannover Medical School, 30625 Hannover, Germany; 8 Department of Urology, University Hospital Münster, 48149 Münster, Germany

**Keywords:** Renal cell carcinoma, CRP, Chromophobe histology, Survival, Biomarkers, Cancer, Oncology

## Abstract

C-reactive Protein (CRP) has been established as a prognostic biomarker in various malignancies, with elevated serum levels correlating with poorer outcomes. However, the significance of tissue-based CRP expression specifically in chromophobe renal cell carcinoma (chRCC), remains inadequately characterized. This study investigates the potential prognostic relevance of intratumoral CRP expression from a substantial cohort of chRCC patients. We conducted a retrospective analysis of patients who underwent surgical intervention for chRCC. Comprehensive clinical data was collected, and immunohistochemical evaluation of tumor specimens was performed to assess intratumoral CRP expression patterns. The study included 81 chRCC patients, with intratumoral CRP expression identified in 35 cases (43.2%). Statistical analysis revealed no significant correlation between CRP expression status and clinical parameters. While 5-year overall survival (OS) analysis showed no statistically significant difference between CRP-positive versus CRP-negative tumors (86.4% versus 100.0%; *p* = 0.106), overall follow-up demonstrated a significantly higher mortality rate in patients with CRP-positive tumors compared to those with CRP-negative tumors (22.9% vs. 2.2%; *p* = 0.013). Our findings suggest that intratumoral CRP expression in chRCC is not clearly associated with parameters of aggressiveness or survival. Further studies should assess the possible correlation between CRP tissue expression and blood levels. However, these findings should be interpreted with caution given the limited sample size, low event rate, and high loss to follow-up, and are best considered hypothesis-generating.

**Supplementary Information:**

The online version contains supplementary material available at 10.1038/s41598-026-60322-2.

## Objective

Chromophobe RCC (chRCC) is the third most common type of renal malignancies with a prevalence of 5–7%. Most chRCC occur sporadically but germline mutations in *FLCN* (Birt-Hogg-Dubé syndrome) and *PTEN* (Cowden syndrome) genes can lead to higher incidences of chRCC. In general, chRCC have a better outcome than other RCC entities, but in some cases, metastases lead to poor survival^1,2^. The World health organizations’ (WHO) classification of 2022 suggests not to use the WHO grading system for chRCC due to the lack of correlation between grading and outcomes for this RCC type^3^. It is thus crucial to find new prognostic markers for chRCC patients, and C-reactive protein (CRP) is among the suggested targets^4^.

CRP is a pentraxine plasma protein and takes part of acute-phase reactions after inflammation. It opsonizes particles such as dead or damaged cells so that the complement system is activated and macrophages are able to eliminate dead cells and pathogens^5^. CRP plasma-levels rise with acute infection but also with chronic inflammatory processes such as rheumatic or cardiovascular diseases^6,7^. It was discussed that not only the presence of CRP but also its conformations and isoforms may play a role in the functioning of CRP and the inflammatory reaction in acute and chronic processes^8,9^. CRP serum levels are elevated in patients with tumors in general, and higher CRP serum-levels are associated with a progression of the disease. Also, higher CRP levels are associated with higher risks for cancer^10^ and with poorer survival in several cancer types, such as cervical, squamous cell, penile or esophageal cancer^11–13^.

The role of CRP was already investigated in clear cell renal cell carcinoma (ccRCC). There, CRP was identified as a prognostic marker and higher serum CRP levels were associated with poorer survival^14–16^. In an analysis of a large cohort of RCC patients, those with a preoperative CRP serum level of 4–10 mg/l and > 10 mg/l had a hazard ratio of 1.66 and 2.58 for cancer related death compared to patients with low perioperative CRP levels^15^. Also, for chRCC, the association between elevated CRP values and all-cause mortality was found, with HR 3.99 (*p* < 0.001)^17^. Not only high serum CRP levels but also CRP tissue expression is associated with worse outcome in some cancer types^12,13^. The role of CRP tissue expression in chromophobe RCC is yet unclear and might qualify as a prognostic marker.

Therefore, the aim of this study was to evaluate the prognostic impact of CRP tissue expression in chRCC on overall survival. To the best of our knowledge, this is the first study which analyzed this aspect in the third most common RCC subtype.

## Material and methods

### Patients and tumor characteristics

Between 1996 and 2014, 81 patients who underwent renal surgery for chRCC were identified through electronic pathology records. All patients were treated at the Klinikum rechts der Isar, Technical University of Munich, Department of Urology. A pathologist (FE) selected appropriate specimens and prepared tissue microarrays (TMAs) from the primary tumors as previously described^18^, with a second uropathologist (AH) confirming the histological subtype. Clinical data for each tissue sample, including tumor stage and histological subtype, were classified according to the UICC 2016 TNM tumor staging system. Patient information, including overall survival (OS), cancer specific survival (CSS) and mortality data, was obtained from electronic medical records and verified through the Munich Cancer Registry of the Munich Tumor Centre. All patients conducted consent to participate. The study was conducted in accordance with the German Human Research Act and the Declaration of Helsinki, with approval from the Ethics Committee of the Technical University of Munich (384/13).

### Procedures

Immunohistochemical (IHC) analysis was employed to evaluate CRP expression. 2 μm TMA slides were stained using a monoclonal antibody against CRP (Clone #IHC-00613, invitrogen, dilution 1:500). The immunostaining protocol consisted of antibody application for 30 min after heat pretreatment at 120 °C for 5 min with Tris–EDTA buffer pH 9 and peroxidase blocking (Dako, Hamburg, Germany). This was followed by incubation with a horseradish peroxidase (HRP)-labeled secondary antibody polymer (EnVision, Dako) for 30, adding a diaminobenzidine (DAB) substrate chromogen solution (Dako) for 10 min and counterstaining for 1 min with hematoxylin (Merck, Darmstadt, Germany). All incubation procedures were performed at room temperature. Positive controls as well as negative control slides without the addition of primary antibody were included for each staining experiment. Paraffin-embedded human liver tissue was included as the positive control. Microscopic assessment was conducted in a blinded fashion by a pathologist (FE), using a Leitz ARISTOPLAN light microscope (Leica Microsystems, Germany) with a × 10 eyepiece, a 22-mm field of view and × 40 objective lens (Plan FLUOTAR × 40/0.70).

The staining reaction was classified according to a semi-quantitative IHC reference scale previously described^19^. CRP showed predominantly cytoplasmatic localization of tumor cells.

**Fig. 1:**
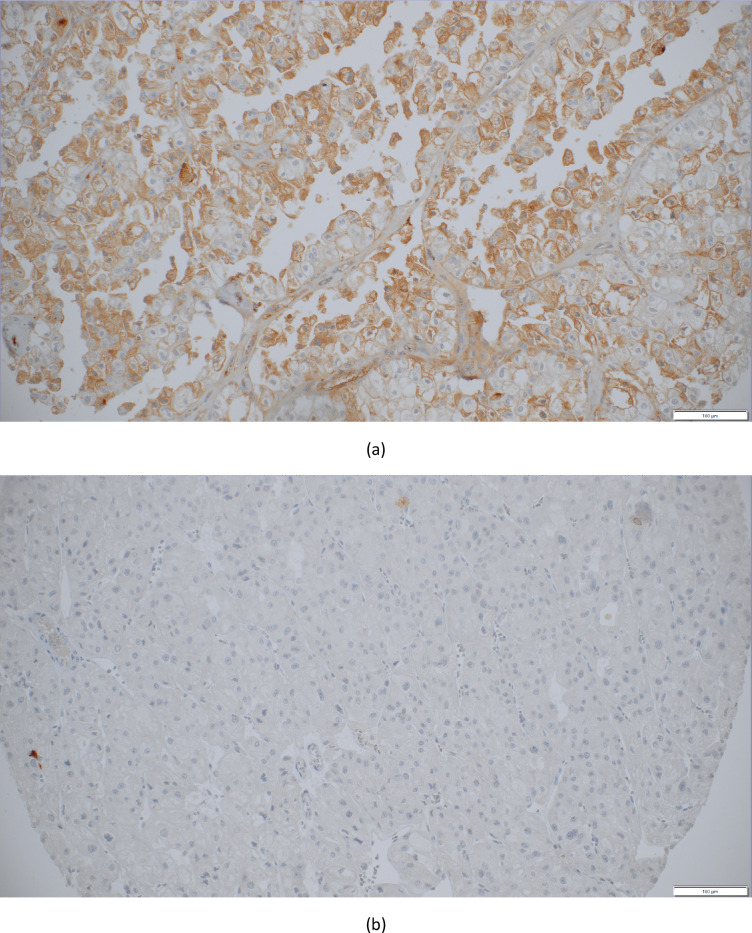
Immunohistochemical staining of CRP in chromophobe renal cell carcinoma specimen. (**a**) positive (40 × magnification) (**b**) negative (40 × magnification).

For semi-quantitative assessment, staining intensity was evaluated using the H-score system ranging from 0 to 3 (0 = no staining, 1 = weak staining, 2 = moderate staining, 3 = strong staining), as illustrated in Fig. 1. The H-score was calculated by multiplying the intensity rating (0–3) by the percentage of stained area (0–100%). Given the lack of established reference values for cytoplasm staining intensity in the literature and our limited sample size, we adopted a binary classification system using the median of observed distributions as the cutoff point, a well-established approach in exploratory immunohistochemical studies. Specimens with CRP staining scores less than or equal to the median (0) were classified as CRP-negative, while those with scores above the median (> 0) were classified as CRP-positive.

### Statistical analysis

Overall survival (OS) was defined as the primary endpoint of this study, and cancer specific survival (CSS) as the secondary endpoint. In the absence of death, the endpoint was censored at the last date of follow-up. Follow-up duration was defined as the interval from the date of surgery to the date of death or last known follow-up. Statistical comparisons between groups were performed using appropriate tests based on data characteristics: chi-square, Fisher’s exact tests, Mann–Whitney U-Test, and independent t-test were applied to compare between patient/tumor characteristics and the corresponding subgroup with or without CRP expression. Survival analysis employed Kaplan–Meier methodology, with subgroups being compared using the log-rank test. First, univariate Cox Regression was performed with Likelihood ratio test. Only the significant covariates and CRP expression were included in the multivariate Cox regression. Two-sided *p* values below 0.05 were considered statistically significant. All statistical analyses were conducted using SPSS 27.0 (USA) and R Studio (version 2024.04.1 + 748) with the packages tidyverse (version 2.0.0) and survival (version 3.7-0).

## Results

### Patients´ characteristics and CRP expression

The study cohort had a median age of 59.8 (interquartile range 52.9–69.1) years. Regarding tumor stage distribution, 60 (74.1%), 14 (17.3%) and 7 (8.6%) presented with pT1, pT2 and pT3 tumors, respectively. The majority of patients (86.4%) had AJCC Stage I/II. Advanced disease (pT3 or pT4 and/or lymph node metastasis and/or synchronous distant metastasis) was observed in 11 (7.4%) patients. Positive CRP expression was found in 35 (43.2%) of the chRCC TMA specimens, respectively (Fig. 1).

No significant associations were identified between CRP + expression and patient or tumor characteristics (Table 1). Male and female participants showed comparable characteristics in terms of age, stage, grade, organ metastases, disease status or CRP expression. However, significantly more women than men had lymph node metastases (3 (13.3%) vs. 0 (0.0%); *p* = 0.021, fisher exact test). There was no difference in 5-year or overall survival between men and women. Gender was not significantly associated with OS or CSS (log-rank *p* = 0.6 and *p* = 1.0, respectively), and CRP expression did not differ significantly between sexes (chi square *p* = 0.203), suggesting that the observed imbalance in lymph node metastases did not confound the survival analyses.

**Table 1:** chRCC patient’s and tumor characteristics in dependence of c-Reactive protein (CRP) expression.

Variable	All chRCC n = 81 (100%)	CRP^−^ n = 46 (56.8%)	CRP^+^ n = 35 (43.2%)	*p* value
Age, median (IQR) years	59.8 (52.9–69.1)	63.7 (52.7–69.2)	57.2 (50.4–69.2)	0.399^a^
Gender				0.144^b^
female	23 (28.4%)	10 (21.7%)	13 (37.1%)	
male	58 (71.6%)	36 (78.3%)	22 (62.9%)	
Stage (TNM 2010)				0.708^c^
pT1	60 (74.1)	33 (71.7%)	27 (77.1%)	
pT2	14 (17.3)	8 (17.4%)	6 (17.1%)	
pT3	7 (8.6)	5 (10.9%)	2 (5.7%)	
Cancer Stage (AJCC)				0.854^c^
Stage I	56 (69.1)	32 (69.6%)	24 (69.6%)	
Stage II	14 (17.3)	8 (17.4%)	6 (17.1%)	
Stage III	8 (9.9)	5 (10.9%)	3 (8.6%)	
Stage IV	3 (3.7)	1 (2.2%)	2 (5.7%)	
LN metastasis^#^				1.0^b^
N-	78 (96.3)	44 (95.7%)	34 (97.1%)	
N +	3 (3.7)	2 (4.3%)	1 (2.9%)	
Metastasis^#^				0.575^b^
M-	78 (96.3)	45 (97.8%)	33 (94.3%)	
M +	3 (3.7)	1 (2.2%)	2 (5.7%)	
Disease status				1.0^b^
Localized *	70 (86.4)	40 (87.0%)	30 (85.7%)	
Advanced ^$^	11 (13.6)	6 (13.0%)	5 (14.3%)	

^#^ at time of renal surgery; * localized disease = pT1/2 N0/M0; ^$^ advanced disease = pT3/4 and/or N + and/or M + . Legend: IQR: Interquartile range, NE: not evaluable; N- = lymph node status unknown or tumour cells absent from regional lymph nodes, N +  = regional lymph node metastasis present. ^a^ Mann–Whitney-U test, ^b^ Fisher exact test, ^c^ chi square test.

### CRP expression and clinical course

Patient follow-up extended for a median duration of 40.5 (IQR: 10.8–109.3) months. At the time of last follow-up, 46 (56.8%) patients were alive, 9 (11.1%) patients died and 26 (32.1%) patients were lost to follow up. When analyzing outcomes by CRP status, in the subgroups of CRP- vs. CRP + 29 (63.0%) vs. 17 (48.6%) patients were alive, 1 (2.2%) vs. 8 (22.9%) patients had died and 16 (34.8%) vs. 10 (28.6%) patients were lost to follow up (*p* = 0.013, chi square).

**Fig. 2:**
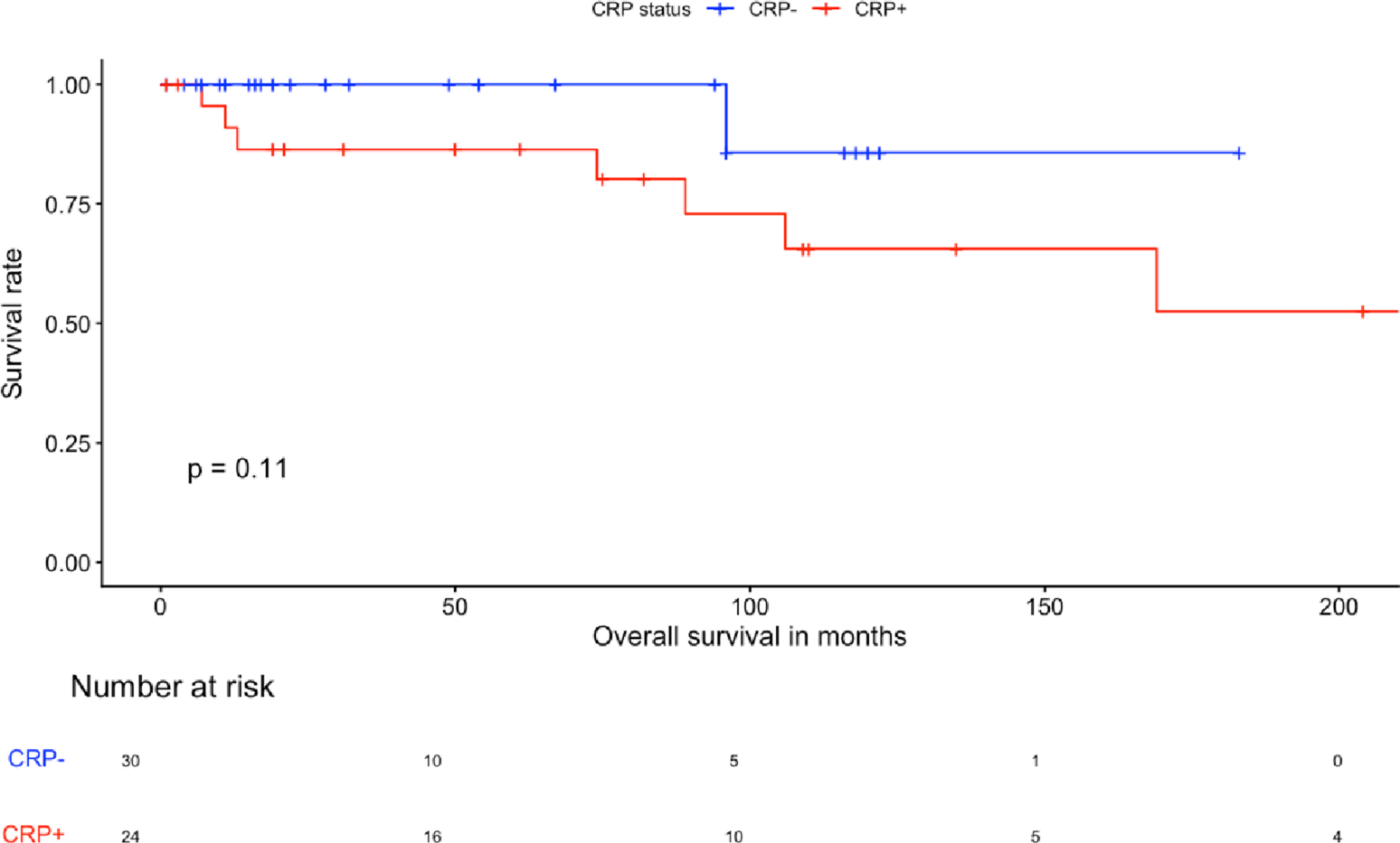
Overall survival for patients with chromophobe renal cell carcinoma in dependence of CRP expression (Kaplan–Meier analysis).

Survival analysis revealed that Kaplan–Meier curves demonstrated a 5 year-OS for CRP- compared to CRP + tumors of 100.0% compared to 86.4% (*p* = 0.106, log rank) (Fig. 2). CSS analyses yielded concordant results, with no statistically significant difference between CRP- and CRP + tumors (*p* = 0.16, log rank; supplementary Fig. 1).

In univariate Cox regression analysis, age was significantly associated with OS (HR = 1.11 (95% CI 1.01–1.22) per year, *p* = 0.026) and CSS (HR = 1.13 (95% CI 1.01–1.27) per year, *p* = 0.008), while CRP expression showed a trend toward adverse outcome that did not reach statistical significance (HR = 4.85 (95% CI 0.59–40.13), *p* = 0.082 for OS, and HR = 4.12 (95% CI 0.48–34.99) per year, *p* = 0.135) for CSS). Gender, AJCC staging, T-, N-, and M-status were not significantly associated with OS. In a subsequent multivariate Cox regression model including CRP expression and age, age remained an independent predictor of OS (HR = 1.10 (95% CI 1.01–1.20) per year, *p* = 0.035) and CSS (HR = 1.12 per year (95%CI 1.00–1.24), *p* = 0.048), while CRP expression did not reach statistical significance (HR = 4.27 (95% CI 0.50–36.37), *p* = 0.185 for OS and HR = 3.49 (95% CI 0.39–31.11), *p* = 0.263 for CSS). In a sensitivity analysis treating CRP H-score as a continuous variable, a consistent trend was observed (OS: HR = 1.010, 95% CI 1.000–1.020, *p* = 0.008; CSS: HR = 1.009, 95% CI 0.998–1.020, *p* = 0.150).

## Discussion

In the new WHO renal tumor classification of 2022, it was advised not to use the WHO grading system for chRCC, because grades and outcomes are not correlated ^3^. Since metastases are rare but associated with higher tumor specific death rates, new prognostic factors to identify patients at risk are warranted. Therefore, we analyzed the role of tumor CRP expression in survival of chRCC patients. Our analyses showed no association of CRP tumor tissue expression with patient or tumor characteristics or 5-year survival. Still, at the time of last follow-up, more patients with CRP^+^ vs. CRP^-^ tumors had died (22.9% vs. 2.2%; *p* = 0.013). That might be an effect of the relatively small cohort with few events. Women had more often lymph node metastases, but there was no difference in survival compared to men.

Regarding the biological validity of CRP expression as a prognostic marker, intratumoral CRP staining intensity has previously been associated with adverse outcomes in other solid tumors, including esophageal carcinoma^13^. Furthermore, tumor CRP expression area has been shown to correlate with serum CRP levels and inversely with prognosis^20^. Importantly, in ccRCC specifically, intratumoral CRP staining intensity has been used as a categorical prognostic variable, with high-intensity staining associated with a markedly increased risk of overall mortality compared to low-intensity staining, independent of serum CRP levels^15^.

Unlike ccRCC, which typically harbors a highly immune-infiltrated tumor microenvironment with abundant CD8 + T cells, tumor-associated macrophages, and regulatory T cells, chRCC is characterized by a markedly immune-cold microenvironment with profoundly depleted T-cell infiltration and reduced expression of HLA class I molecules^21^. In ccRCC, CRP has been shown to interact with CD64-expressing macrophages, promoting IL-6 secretion and PD-L1 upregulation, thereby contributing to an immunosuppressive environment linked to adverse outcomes^22,23^. In chRCC, however, the relative paucity of immune cell infiltrates—including macrophages—may limit the functional relevance of CRP-mediated inflammatory signaling within the tumor. Furthermore, chRCC exhibits distinct metabolic features, including mitochondrial dysfunction and altered lipid metabolism, which may shift oncogenic pathways away from the inflammation-driven mechanisms through which CRP exerts its prognostic effects in other tumor entities. Taken together, these biological differences suggest that the CRP-inflammation axis, while prognostically relevant in ccRCC, may be of limited significance in the immunologically quiescent context of chRCC.

In other tumor entities and in chRCC, the association between high CRP serum levels and poor outcome could be shown, since higher CRP levels hint to an advanced disease status^17,24^. Nonetheless, CRP serum levels might be caused by local inflammation, since CRP tumor expression has been shown to correlate with CRP serum levels in ccRCC^25^. In this context, it is important to define what to measure since CRP levels in serum are highly variable and absolute values might be organ-specific. For example, patients with gastric tumors show higher CRP levels than patients with renal cancer^26^. Hart et al. suggest maximum CRP levels or CRP/albumin ratio as an alternative measure^26^. Additionally, CRP polymorphisms or isoforms might characterize the disease status more adequately. Monomeric CRP is highly bioactive and occurs mostly in acute infections, whereas pentameric CRP is less bioactive and hints to an ongoing disease such as cancer^9^. The more CRP polymorphisms were present, the higher was the CRP expression of the tumor^27^. In this study, we did not measure serum levels or CRP polymorphism, but the lacking association between CRP tumor expression and survival hints to a need in comprehensive research with comparison on different CRP modalities.

While a positive correlation between serum and tissue CRP levels has been linked to an immunosuppressive microenvironment and adverse prognosis in ccRCC^25^, this relationship has not been established for chRCC. Notably, serum CRP has been shown to be prognostically significant in chRCC^17^, suggesting that the systemic inflammatory response may carry prognostic information in this entity even when local tissue expression does not. The discordance between our tissue-based findings and previously reported serum-based associations underscores the possibility that serum CRP in chRCC may primarily reflect tumor burden or host-related factors rather than local inflammatory activity within the tumor itself. Future studies integrating both tissue and serum CRP data in chRCC cohorts are warranted to clarify this relationship.

While the present study did not include treatment-related data, the broader literature suggests potential clinical applications of CRP monitoring that warrant investigation in future studies. Not only the survival of patients with high CRP serum levels is worse, also the response on tyrosine kinase inhibitor therapy is impaired^28^. A recent investigation showed that CRP kinetics might predict response to immunotherapies, with higher CRP serum-levels indicating worse progression-free survival^29^. Not only the course of serum CRP levels, but also a higher CRP level at initiation of checkpoint inhibitor therapies is associated with poorer overall survival^30^. As discussed above CRP activates macrophages via the CRP/CD64 axis^22^ and CRP kinetics may thus serve as a tool to evaluate tyrosine kinase or checkpoint inhibitor therapy. Not only cancer-specific survival is associated with higher CRP levels, also kidney function and with-it morbidity is worse in RCC patients with higher CRP levels^31^. The reason for an impaired survival might also be caused by comorbidities, that elevate the CRP serum level. In some experimental studies in patients with acute coronary ischemia or COVID-19, serum CRP levels were decreased with aphaeresis and those patients showed good outcomes^32,33^. Apart from those experimental studies, CRP was not considered as a target for specific therapy.

In our cohort, more women had lymph node metastases than men, but the survival did not differ between the groups. According to the literature, the prevalence of RCC in men in higher, but women have a higher mortality in metastatic RCC^34^, which we could not confirm with our data.

This study has some limitations that warrant consideration. First, the number of cases and events is relatively small due to the low prevalence of the disease, representing 5% of all RCC cases. Published cohorts with pathological and IHC features report similar sizes^35^ which reflects the small overall prevalence. Furthermore, dichotomization of CRP expression at the median H-score may not represent the optimal threshold. The continuous Cox analysis yielded consistent results, supporting the robustness of our findings. Due to the limited number of events, the multivariate model was restricted to CRP status and age as the strongest clinical confounder, precluding a fully adjusted analysis. Therefore, all survival analyses, including the multivariate model, should be considered exploratory and interpreted with caution. Second, a third of the patients was lost to follow-up, which might lead to an attrition bias. However, patients lost to follow-up did not differ significantly from those who completed follow-up in age, gender, T-, N-, M-status, or AJCC staging. The event rate observed in this cohort is consistent with the generally favorable prognosis of chRCC, and the survival analyses should be interpreted within this epidemiological context. Furthermore, the follow-up period ranged up to 226 months, reflecting the protracted timelines required to accrue survival events in this indolent entity. Nevertheless, the modest median follow-up of 40.5 months should be considered when interpreting survival estimates, particularly for five-year overall survival analyses.

chRCC is a heterogenous tumor type with small cell subtypes^36^, which might lead to underestimation of the impact of CRP. We performed retrospective analyses, used TMAs and methodology, immunohistochemistry and the interpretation system can be improved. Also, there were no serum values for CRP available.

In summary, we assessed the prognostic role of CRP tissue expression in a large cohort of chRCC patients. CRP expression was not associated with tumor or patients’ characteristics or survival in our cohort. Nonetheless, further research regarding tissue CRP with serum levels for validation as prognostic marker for chRCC is warranted.

## Supplementary Information

Supplementary Information.

## Data Availability

The data that support the findings of this study are not openly available due to reasons of sensitivity and are available from the corresponding author upon reasonable request.

## Abbreviations

AJCC: American Joint Committee on Cancer
chRCC: Chromophobe renal cell carcinoma
CRP: C-reactive protein
HRP: Horseradish peroxidase
IHC: Immunohistochemistry
ISUP: International Society of Urological Pathology
OS: Overall survival
RCC: Renal cell carcinoma
TMA: Tissue micro arrays
WHO: World Health Organization
