# Supplementary material for: Evaluating the clinical significance of tumor-expressed C-reactive protein in chromophobe renal cell carcinoma
Source: Sci Rep. 2026 Jul 14;16:22077. doi: 10.1038/s41598-026-60322-2 (PMC13370012; doi:10.1038/s41598-026-60322-2)

# *Supplementary Figure 1: Cancer specific survival for patients with chromophobe renal cell carcinoma in dependence of CRP expression (Kaplan-Meier analysis)*

#
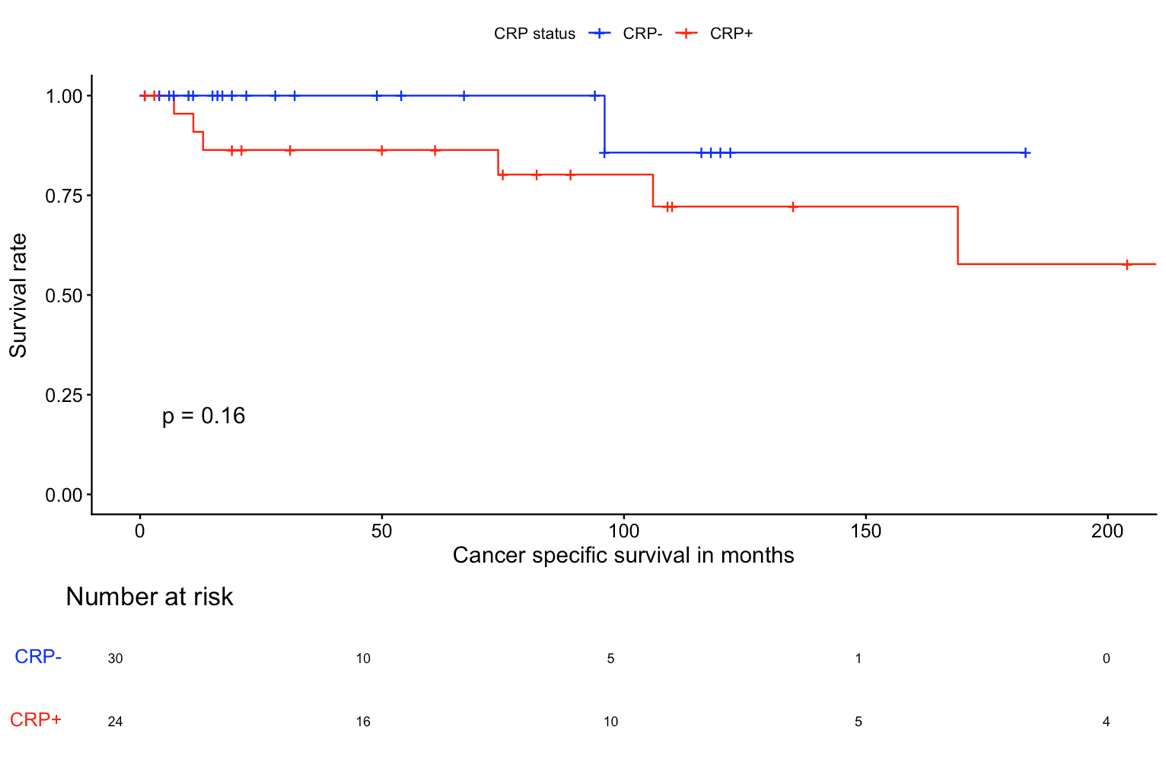

Supplement: Supplementary file 1 — Supplementary Information. [file 41598_2026_60322_MOESM1_ESM.docx]
